# Policy critique: The conflation of shaken baby syndrome and abusive head trauma – a measure with several negative effects

**DOI:** 10.1016/j.fsisyn.2025.100585

**Published:** 2025-04-11

**Authors:** Niels Lynøe, Anders Eriksson

**Affiliations:** a Centre for Healthcare Ethics, Karolinska Institutet, Stockholm, Sweden; b Department of Clinical Sciences, Forensic Medicine, Umeå University, Umeå, Sweden

## Abstract

The amalgamizing of shaken baby syndrome (SBS) with the much broader and heterogeneous abusive head trauma (AHT) diagnosis is problematized. We suggest that the reason why American Academy of Pediatrics (AAP) took this step in 2009 was a response to another theory being presented and discussed from 2001 and onwards. This theory had completely different legal consequences as it suggested that the medical findings on which the SBS diagnosis were based, i.e. “the triad” (subdural hemorrhages/SDH, retinal hemorrhages/RH, and encephalopathy) were non-traumatic. If such an explanation was accepted, this would reveal not only that serious legal abuses had occurred in the past and that the pediatricians should be held responsible for this, but also that it would in the future be more difficult to protect the child by claiming abuse in cases of unclear diagnosis. We present also other steps, taken by other pediatric organizations, having similar effects upon the current SBS controversy.

We suggest that these value-based considerations were the underlying reasons why SBS was integrated in the AHT concept, and why competing theories and evidence-based criticism is ignored, allowing to always interpret triad findings as the result of abuse. If the ethical principle to protect the child is more important to AAP than the scientific ambition to develop evidence-based diagnostic procedures, we encourage AAP to be honest and admit this prioritization. Or at least to admit that in this ethical dilemma, AAP finds that the least bad choice is *First of all, protect the child!* despite the price is that many infants and its siblings may be separated on wrong grounds from their family, and that caregivers might be falsely accused and convicted of child abuse.

## 1 Introduction

### 1.1 Two competing theories

The uncontested explanation of the combination of subdural hemorrhage (SDH), retinal hemorrhages (RH), and encephalopathy – so-called triad findings - without signs of relevant trauma was until two decades ago *violent shaking*. This interpretation was summarized in the term Shaken Baby Syndrome (SBS), thus forming the traditional SBS theory. In the early 2000's another theory was presented [1,2], suggesting a non-traumatic explanation of triad findings and hence challenging the need of violent shaking. This “unified theory” suggested that hypoxia triggered a cascade of events: brain edema, raised intracranial pressure, oozing of blood from small vessel walls, and hence the triad findings (2). The primary cause of hypoxia was suggested to be a spontaneous breathing stop and lifelessness attack, caused by, e.g., the infant's immature brain [3]. The background of this theory was that autopsies of suspected SBS cases showed no disrupted nerve fibers as expected according to the traditional SBS theory [4] - by contrast neuropathology analyses disclosed severe hypoxic brain swelling [1].

Instead of attracting interest among concerned pediatricians and researchers, the “unified theory” was soon criticized and ignored [5,6]. Although the authors responded to the criticism and found that it was based merely on misunderstandings [7], the theory was considered as an unverified hypothesis, and was not allowed to be presented in courts in the UK [6]. Even though the hypoxia theory must be questioned and discussed - like all new hypotheses and mechanism theories – it deserves to be systematically tested. This was never done, apart from one study – using Hills criteria for assessing causality [8] – in which the hypoxia theory was found more plausible than the traditional SBS theory [9]. This was however ignored, and it was instead falsely claimed that the first author had withdrawn her hypothesis [10].

As nobody reacted officially to the dismissal of a non-tested theory, we began to wonder if there were also non-scientific reasons for this dismissal. One such possibility is the different legal consequences of alternative, non-traumatic theories. If a non-traumatic theory was tested and found evidence-based, this could be used to question the diagnostic procedure in suspected SBS: Hence, the legal consequences could be that a father accused of violent shaking could be acquitted. This consequence could also counteract the ethical principle, *First of all, protect the child*, and thereby considered unacceptable to the advocates of the traditional SBS theory.

## 2 First step taken by the American academy of pediatrics

Without testing the non-traumatic theory, the AAP amalgamated the SBS diagnosis into the wider and heterogeneous concept of Abusive Head Trauma (AHT) in 2009 (11). In this way, a crack-free AHT wall appeared to have been established [11,12]. In addition, by introducing the term “abuse”, it was now not only implied that isolated triad findings were caused by shaking, it was also implied that there was criminal intent behind the alleged shaking. No room was left for other explanations, e.g., revival shaking or non-traumatic mechanisms. Notably, such a conflation of two different conditions - *with* and *without* signs of trauma, respectively, and thus with potentially different pathogenesis - constitutes a step in the opposite direction from the norm in medicine, i.e. increased differentiation for the benefit of increased understanding and treatment.

From this 2009 statement onwards, any pediatrician could be confident that *all* triad cases would be considered to have been subjected to abuse, thereby avoiding being blamed for false accusations. Any case where a child protection team concluded that shaking had caused the triad findings, would now lead to charges of assault – or worse. An epidemiological study of infant mortality in the US 1940–2005 showed that from around 1980, accidental deaths decreased in number, while homicides increased to a corresponding extent [13]. The authors suggested that this converse relationship was linked to increased societal sensitivity towards child abuse, rather than an actual increase in societal violence directed against infants, which biased the classification. Hence, if there was an increase in incorrectly suspected homicides, the number of caregivers who considered themselves innocently accused would also increase. Also defense lawyers and researchers began to question the scientific basis of the SBS diagnosis. Eventually, this contributed to an independent Health Technology Agency (“SBU”) undertaking a systematic review of the scientific evidence of triad findings for diagnosing SBS [14]. The results revealed that almost all publications on which the SBS diagnostic procedure rested were affected by incorporation bias and circular reasoning - and thus assessed as having very low scientific quality.

## 3 Harsh criticism of evidence-based conclusions

Relevant in this context are, above all, the feared legal consequences of the conclusions of the SBU report, but also the relevance of “the triad” was questioned.

- A group of critical English authors (on behalf of the Royal College of Paediatrics and Child Health) stated that the SBU report had the “potential to undermine medico-legal practice”, represented “pseudoscience”, and should be retracted [15].
- Another group of critical authors (representing the European Society of Radiology) stated that the SBU report, if unchecked, “will result in failure to protect abused and vulnerable children”. Further, the evidence-based results in the SBU report “appears to be directly borrowed from the lawyers …“, “providing lawyers with new ammunition to question valid scientific data”, and may have “catastrophic consequences”. They also stated that the triad “is a lawyer-created name” [16].
- A group of critical French authors (with high positions in Haute Autorité de Santé, which issues clinical guidelines) complained that the SBU report “is already being used by defense lawyers”, and “must be ignored” [17].
- In a so-called consensus document (endorsed by a number of national pediatric and radiological societies), the SBU report was criticized, and stated to be “likely to confuse judges and juries” [18]. Furthermore, one of its authors stated in a separate article that they “revealed severe prejudicial bias” in the SBU report (19), and concluded that “this alternative agenda has no role in true science and can result in infant harm through shaking and neglect …“.

## 4 First of all, protect the child!

As noted, the wordings are emotionally charged, and indicate that the starting points were more value-based than scientific. On the contrary, the methodology used in the SBU report is scientifically well established and reported in detail. However, these critical comments seem to have a common denominator, focusing on threats towards the above-mentioned ethical principle *First of all, protect the child!* The significance of this ethical principle and its influence when classifying suspected child abuse was supported not only by the epidemiological study [13], but also by the emotional exclamation “it is NEVER acceptable to shake a baby!” [20] as a rebuttal of scientifically relevant questions; it goes without saying that no one has claimed the opposite, nor argued against the ethical principle. But ethical principles must not be hidden as apparent scientific reasoning.

The statements that the SBU report will “confuse judges and juries” and have “catastrophic consequences” mean that if the diagnostic accuracy of traditional SBS diagnosis must become evidence-based rather than value-based, the anticipated effect would be that these infants can no longer be protected from feared abuse. The claims that the SBU report suffered from “severe prejudicial bias”, represent “pseudoscience”, and that this “alternative agenda has no role in true science” act as a plea to overlook the evidence-based results in SBU report – and even to demand it retracted [15] or ignored [17]. This is perhaps understandable if an inevitable consequence of the report were future abuse. But what if the consequence instead is that an evidence-based investigation procedure can discriminate between shaken and non-shaken babies? If so, this might prevent infants and its siblings from being separated from its family without reason, and prevent innocent caregivers from being falsely accused of child abuse. From scientific, medical, societal, social, and legal standpoints, this would be a considerably better solution.

## 5 The ethical dilemma

However, according to a pediatric surgeon who was interviewed in Swedish public service television in 2021, the risk of false accusations, wrongful convictions, and baseless splitting of families is the price which must be paid by children and caregivers to *protect the child!* [21]. This pediatrician might have concluded that false accusations represent the least bad choice in this ethical dilemma. At least he was honest what it is all about – not insinuating that there was a hidden agenda behind the SBU report or that it represented “pseudoscience”.

## 6 Triad findings

Moreover, the claim that the term triad is “lawyer-created” [16] is probably a value-impregnated claim, which surprisingly contrasts with the statement “the medical fact that the diagnostic triad … can reliably be associated with abusive head trauma” in the same publication [16]. It also ignores the fact that “triad findings” have been used as an allegedly safe diagnosticum for decades. In fact, in many countries it was not until the SBU report revealed the shortcomings of this widely used triad diagnosis that it became uncomfortable to use this term. Triad findings are however still used in clinical guidelines [e.g. 22], applied by researchers as both diagnostic test and reference standard [23,24], and used in criminal and civil court proceedings as the basic evidence of intent and guilt [25]. Furthermore, in 2016 one of the co-authors of the “consensus document” presented a study which disclosed the wide acceptance of the triad in the diagnostic process of AHT [26]. Hence it seems more probable that not only prosecutors but also defense lawyers had learned about the alleged specificity and practical importance of triad findings from medical expert witnesses representing child protection teams, long before the publication of the SBU report!

One effect of the SBU report is, however, that the importance of triad findings is now generally denied by child abuse pediatricians, and instead the concept of “the whole picture” was introduced and listed as the diagnostic tool. If, however, “the whole picture” is used as a diagnostic test, there is no reference standard to be used except the *identical* test, viz. “the whole picture”! But if the diagnostic test and the reference test are identical, then we are again in the same situation that has characterized this entire field of research from the very beginning, i.e. incorporation bias and circular reasoning. Although it may appear as if circular reasoning results in new knowledge, it is in the present context like presenting a new hypothesis which is not falsifiable [27]. In other words, use of “the whole picture” as a diagnostic (and reference) test is a safe indicator of *true* pseudoscience.

Although some critics of the SBU report [19,28] suggested that circular reasoning must be accepted to a certain degree when diagnosing SBS, this is again to accept that the SBS diagnosis is more value-based than evidence-based. However, such a convoluted explanation as “the whole picture” is clearly necessary if the higher goal and preference is to be achieved. Moreover, the composition of a multidisciplinary team may, in addition to medical personnel and representatives from social services, include also a clergy [18]. Is the role of a clergy in such a team - who makes the SBS diagnosis based on a “medical conclusion” - to ensure that the above-mentioned ethical principle is guaranteed?

## 7 Concluding remarks

In summary, the various steps and measures described all aim to prioritize value-based opinions over evidence-based conclusions. Some critics of the SBU report stated that the report represented “pseudoscience” and should be retracted [15]. It is tempting to use similar prompts to AAP concerning its 2009 and 2020 policy documents [11,12] where suspected SBS is conflated into the broad and heterogeneous group of AHT, with potentially very different pathogenetic mechanisms. We abstain, however, from this option and instead suggest that AAP is honest and admits - like the Swedish pediatric surgeon - that their belief in this ethical dilemma is that the risk of removing children from a well-functioning family, and of falsely accusing a caregiver of child abuse is the price which must be paid for prioritizing the ethical principle *First of all, protect the child!* This ethical preference is, however, now disguised in seemingly scientific reasoning. If admitting so it would no longer be necessary to amalgamize suspected SBS into AHT. AAP could also stimulate evidence-based research regarding triad findings as a diagnostic test and perhaps eventually identify an appropriate reference standard by which to optimize the diagnostic accuracy of what is currently referred to as SBS and what is not.

## CRediT authorship contribution statement

**Niels Lynøe:** Writing – original draft, Conceptualization. **Anders Eriksson:** Writing – review & editing, Visualization, Validation.
